# Needle Tract Seeding Following Transgastric Endoscopic Ultrasound‐Fine Needle Aspiration for Pancreatic Tail Cancer: A Case Report

**DOI:** 10.1002/deo2.70243

**Published:** 2025-12-26

**Authors:** Hidetaka Kuroda, Takuji Iwashita, Yosuke Ohashi, Shota Iwata, Ryuichi Tezuka, Shinya Uemura, Katsutoshi Murase, Nobuhisa Matsuhashi, Tatsuhiko Miyazaki, Masahito Shimizu

**Affiliations:** ^1^ First Department of Internal Medicine Gifu University Hospital Gifu Japan; ^2^ Department of Gastroenterological Surgery/Pediatric Surgery Gifu University Hospital Gifu Japan; ^3^ Department of Pathology Gifu University Hospital Gifu Japan

**Keywords:** EUS‐FNB, EUS‐FNA, needle tract seeding, pancreatic cancer, transgastric puncture

## Abstract

We report the case of a 69‐year‐old man with a branch‐duct intraductal papillary mucinous neoplasm who underwent surveillance endoscopic ultrasound (EUS), which revealed a 15 mm hypoechoic mass in the pancreatic tail. Transgastric EUS‐guided fine needle aspiration (FNA) confirmed a diagnosis of resectable pancreatic ductal adenocarcinoma. After neoadjuvant chemotherapy, the patient underwent laparoscopic distal pancreatectomy. Twelve months later, contrast‐enhanced computed tomography demonstrated a 20 mm mass lesion in the gastric body. Esophagogastroduodenoscopy showed a submucosal tumor‐like lesion, and endoscopic biopsy from the lesion confirmed adenocarcinoma. Laparoscopic local gastric resection was performed, and needle tract seeding (NTS) was diagnosed based on clinical and histopathological findings. Four months after gastric surgery, peritoneal dissemination was suspected, and chemotherapy with gemcitabine plus nab‐paclitaxel was initiated. This case highlights the risk of NTS after transgastric EUS‐FNA and underscores the importance of careful postoperative surveillance.

## 1 Introduction

Endoscopic ultrasound‐guided fine‐needle aspiration (EUS‐FNA) is an ultrasound‐guided procedure that allows access to lesions near the gastrointestinal tract. It is widely used in general clinical practice to obtain pathological specimens, particularly for diagnosing pancreatic cancer. EUS‐FNA is a minimally invasive and safe method, and its importance is increasing with the expanding use of preoperative chemotherapy in resectable or borderline resectable pancreatic cancer. However, there have been increasing reports of needle tract seeding (NTS) after EUS‐FNA. Here, we report a case of NTS.

## 2 Case Report

**FIGURE 1:**
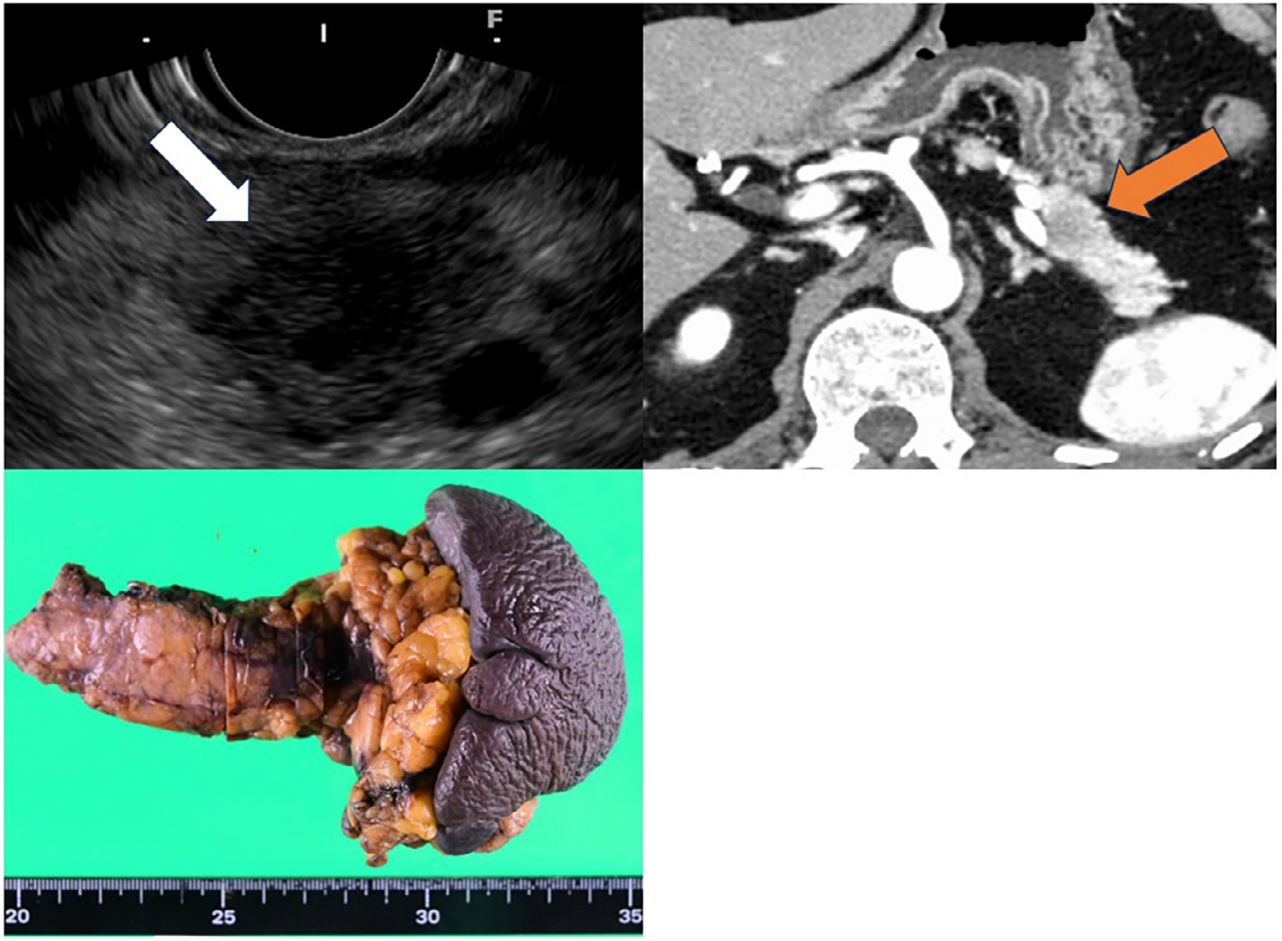
(Upper left) Endoscopic ultrasound showed a 15 mm hypoechoic mass (arrowheads) in the pancreatic tail. (Upper right) Contrast‐enhanced computed tomography showed a 15 mm hypo‐enhanced mass (arrow) in the pancreatic tail. (Lower left) The resected specimen of laparoscopic distal pancreatectomy.

A 69‐year‐old man with branch‐duct intraductal papillary‐mucinous neoplasm (BD‐IPMN) in the pancreatic tail had been under surveillance with periodic imaging and blood tests since August 2021. Magnetic resonance cholangiopancreatography showed no abnormal findings other than BD‐IPMN in 2022. Six months later, surveillance EUS revealed a 15 mm hypoechoic mass in the pancreatic tail, although no change was observed in the BD‐IPMN in the pancreatic tail (Figure 1, Upper left). Blood tests also revealed elevated tumor markers, including CEA (5.6 ng/mL) and CA19‐9 (155.0 ng/mL). Contrast‐enhanced computed tomography (CT) also revealed a 15 mm hypoenhanced mass in the pancreatic tail (Figure 1, Upper right). Transgastric EUS‐FNA of the pancreatic tail mass was performed using a 19‐gauge needle (SonoTip TopGain: Medi‐Globe, Rohrdorf, Germany). Two passes were made, with approximately 10 to‐and‐fro movements per pass, and 10 mL of negative pressure was applied. Pathology demonstrated adenocarcinoma. Positron emission tomography (PET)‐CT revealed increased FDG uptake (SUV max: 3.66) in the pancreatic tail mass, with no other abnormal uptake. The diagnosis was resectable pancreatic ductal adenocarcinoma, clinical stage IA (T1cN0M0, UICC eighth). Pancreatic cancer was not associated with the BD‐IPMN.

The patient received two courses of neoadjuvant gemcitabine plus S‐1 chemotherapy, followed by laparoscopic distal pancreatectomy (DP) and lymphadenectomy. No peritoneal dissemination or other organ invasion was seen intraoperatively, and resection was completed without complications. Pathology revealed a 20 × 15 × 10 mm whitish mass with tubular growth of atypical epithelium and fibrosis, consistent with well to moderately differentiated adenocarcinoma. There was involvement of peripancreatic fatty tissue and veins, but no lymphatic invasion, perineural invasion, extension into the main pancreatic duct, or invasion into the spleen. The resection margins were negative, but there was one metastasis in a regional lymph node. Immunostaining showed p53 wild or null pattern, CK7(+), CK20 (weak+), MUC5AC (partial+), and MUC6 (partial+) (Figure 1, Lower left). The final diagnosis was pancreatic ductal carcinoma, stage IIB (T1cN1M0). After surgery, S‐1 monotherapy was performed for 6 months as adjuvant chemotherapy.

**FIGURE 2:**
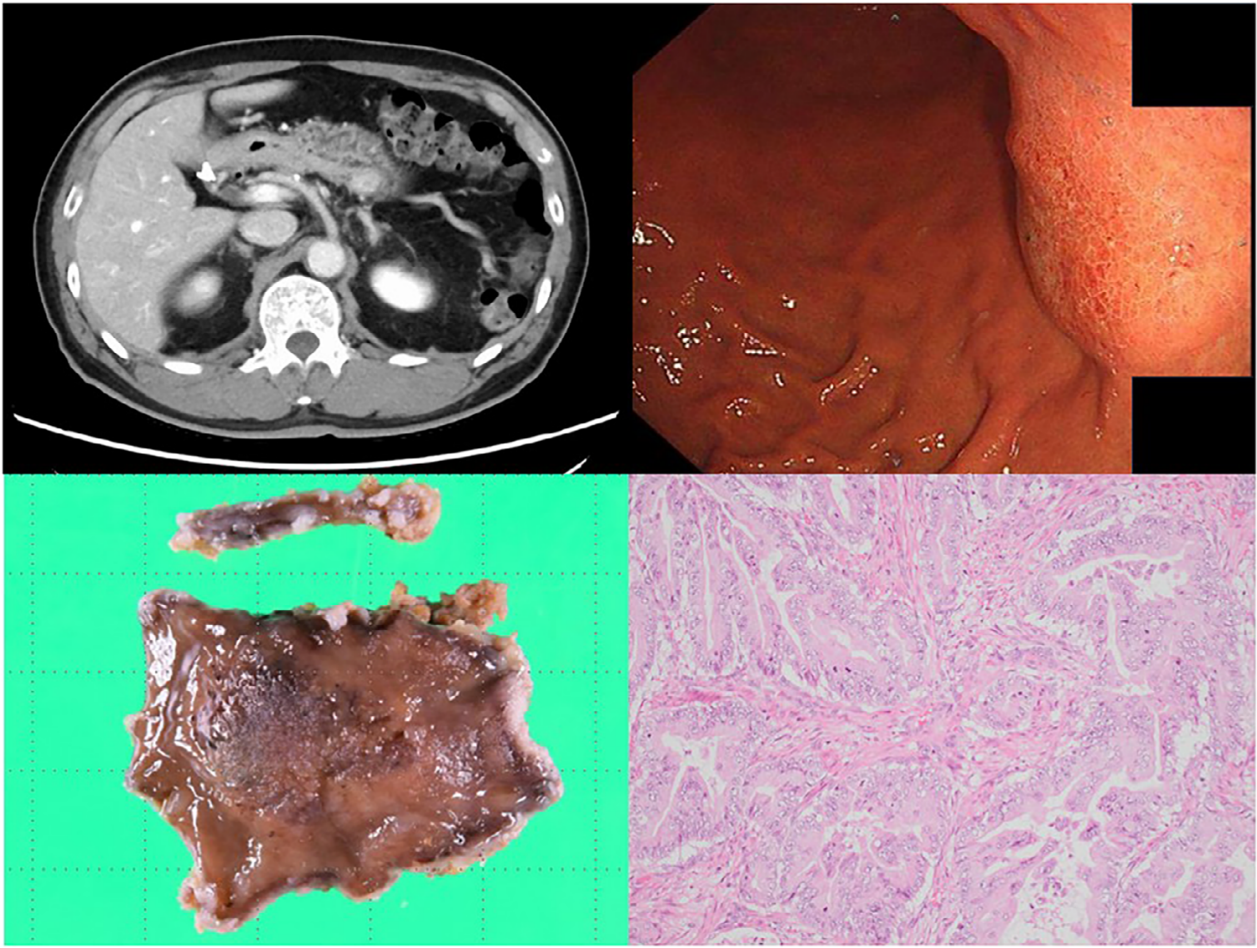
(Upper left) A mass lesion of about 20 mm (arrow) in the gastric wall was recognized. (Upper right) Upper gastrointestinal endoscopic image, showing a 30 mm submucosal tumor‐like bulge on the posterior wall of the gastric body. (Lower left) Atypical cells are infiltrating and proliferating, forming fused ductal structures. (Lower right) Histopathological findings of a gastric biopsy. Hematoxylin and eosin (H&E) staining, medium power (×10 objective; total magnification ×100).

One year after surgery, postoperative surveillance using contrast‐enhanced CT revealed a 20 mm mass located in the posterior wall of the stomach (Figure 2, Upper left), with CA19‐9 elevated to 214 U/mL. PET‐CT showed low‐grade uptake in the gastric body with no abnormal uptake elsewhere. Esophagogastroduodenoscopy (EGD) revealed a 30 mm submucosal lesion on the posterior wall of the gastric body (Figure 2, Upper right). The covering mucosa appeared erythematous and finely granular on NBI magnification, which was not a typical finding of gastric cancer. Endoscopic biopsy revealed adenocarcinoma, with immunostaining showing a p53 wild pattern, MUC5AC (+), and MUC6 (focal +) (Figure 2, Lower right). The pathological features were similar to those of the resected pancreatic cancer, and the gastric lesion was highly suspicious for NTS.

The patient underwent laparoscopic local gastrectomy 14 months after the initial surgery (Figure 2, Lower left). There was no obvious dissemination in the abdominal cavity. The gastric lesion was resected laparoscopically with the assistance of intraoperative EGD to confirm the tumor margins. Pathological examination of the locally resected gastric specimen revealed atypical cells infiltrating and proliferating with fused glandular ducts and cribriform structures, resembling those of pancreatic cancer, and was consistent with moderately differentiated adenocarcinoma. The tumor invaded from the intramucosa to the submucosa, but the superficial layer showed normal gastric mucosa structure. Accordingly, a definitive diagnosis of NTS was established based on the clinical course and surgical pathology.

At 1 month after surgery, contrast‐enhanced CT demonstrated no evidence of recurrence. In contrast, CT performed 4 months after surgery for NTS revealed findings suspicious for peritoneal dissemination. Consequently, chemotherapy with gemcitabine plus nab‐paclitaxel was initiated and continues at 8 months after NTS surgery.

## 3 Discussion

EUS‐FNA is widely used to obtain pathological specimens from pancreatic lesions, particularly in the management of pancreatic cancer [1]. It demonstrates good diagnostic accuracy. While complications such as bleeding, pancreatitis, and pain can occur, their incidence is low (approximately 2.5%), making the procedure generally safe and useful [2]. However, since the first case report of pancreatic cancer following EUS‐FNA by Hirooka et al. [3], the number of reported cases has increased as awareness of NTS has grown (Table 1). Since the diagnostic criteria for NTS have not been clearly established, we defined it as: (1) development of a tumor at the site corresponding to the FNA puncture, and (2) pathological findings similar to the pancreatic primary with exclusion of gastric origin. In this case, the tumor arose at the site corresponding to the FNA puncture route, and the resected specimen revealed pathological features resembling those of the primary pancreatic lesion, consistent with pancreatic ductal adenocarcinoma; therefore, the case was diagnosed as NTS.

**TABLE 1:** The number of cases reported about needle tract seeding (NTS). Abbreviation: DP, distal pancreatectomy.

Author	Year	Age	Sex	Size (mm)	Needle (G)	Initial therapy	Interval from surgery	Examination	Treatment
Paquin SC [4]	2005	65	Male	22	22	DP	21 months	Contrast‐enhanced CT	Chemotherapy
Ahmed K [5]	2011	75	Male	NA	22	DP	39 months	PET‐CT	TG
Chong A [6]	2011	55	Female	27	22	DP	26 months	PET‐CT	NA
Katanuma A [7]	2012	68	Female	20	22	DP	22 months	EGD	NA
Tomonari A [8]	2015	78	Male	25	22	DP	9 months	EGD	SG
Sakurada A [9]	2015	87	Female	25	22	DP	19 months	CA19‐9	Gastrectomy
Minaga K [10]	2015	63	Female	20	22	DP	8 months	CA19‐9	LG
Minaga K [11]	2016	NA	NA	30	NA	DP	24 months	NA	Gastrectomy
Iida T [12]	2016	78	Female	NA	22	DP	6 months	EGD	Chemotherapy
Kita E [13]	2016	68	Female	NA	22	Radiation	7 months	PET‐CT	NA
Yamauchi J [14]	2016	65	Female	25	22	DP	22 months	EGD	LG
Sakamoto U [15]	2018	50	Male	38	22	DP	24 months	EGD	LG
Yasumoto M [16]	2018	78	Female	10	25	DP	22 months	EGD	LG
Matsui T [17]	2019	68	Female	15	19–22	DP	1 month	DP	LG
Matsui T [17]	2019	70	Male	15	22	DP	3 months	DP	LG
Yonemitsu K [18]	2019	62	Male	50	25	DP	13 months	EGD	LG
Kawabata H [19]	2019	78	Female	11	22	DP	24 months	Contrast‐enhanced CT	LG
Sato N [20]	2020	83	Female	26	22	DP	3 months	Contrast‐enhanced CT	LG
Rothermel LD [21]	2020	61	Male	37	25	DP	42 months	EGD	LG
Hayasaka S [22]	2020	75	Female	NA	22	DP	2 months	EGD	LG
Okamoto T [23]	2020	72	Female	42	22	DP	18 days after EUS‐FNA	Contrast‐enhanced CT	DP+LG
Kojima H [24]	2021	81	Male	13	22	DP	NA	Pathological tissue diagnosis	NT
Ogura Y [25]	2021	80	Female	NA	NA	DP	12 months	Contrast‐enhanced CT	LG
Uozumi N [26]	2021	77	Female	32	22	DP	NA	DP	DP
Nagano S [27]	2021	67	Female	NA	NA	DP	34 months	Contrast‐enhanced CT	LG
Yane K [28]	2022	82	Male	22	22	DP	42 days after EUS‐FNA	Contrast‐enhanced CT	DP+LG
Archibugi L [29]	2022	57	Male	20	25	DP	7 months	Contrast‐enhanced CT	LG
Yoshida E [30]	2023	70	Female	70	22	DP	21 days after EUS‐FNA	Pathological tissue diagnosis	Chemotherapy

A nationwide survey in Japan reported NTS in 40 (0.33%) of 12,109 EUS‐FNA procedures for primary pancreatic tumors performed at 235 institutions between April 2010 and March 2018 [31]. Of these, 38 cases (0.409%) occurred after EUS‐FNA for pancreatic cancer. Importantly, all 38 cases were associated with transgastric puncture, whereas no cases were reported following transduodenal puncture. The absence of reported NTS after transduodenal puncture may be explained by the fact that this approach is mainly used for pancreatic head tumors, and the puncture site in the duodenum is resected. Ngamruengphong et al. reported no difference in the risk of postoperative gastric wall recurrence or peritoneal dissemination after preoperative EUS‐FNA for pancreatic cancer [32]. However, they suggested that NTS occurs with a certain frequency, primarily after transgastric EUS‐FNA for pancreatic cancer treated by DP.

Currently, there is no established diagnostic method for NTS. NTS has been detected through imaging studies, such as contrast‐enhanced CT and PET‐CT, during post‐pancreatectomy surveillance [4, 5, 6, 13, 19, 20, 23, 24, 25, 27, 28, 33], or identified as a gastric mucosal tumor‐like lesion during EGD [7, 8, 11, 12, 14, 15, 16, 18, 21, 22]. In some cases, NTS has been detected during further examinations prompted by elevated CA19‐9 levels [9, 10]. In previous reports, the median interval from surgery to NTS diagnosis was 19 months (range, 2–42 months)^1^. There are also reports of patients with pre‐operative CT findings suspicious for NTS who underwent gastrectomy concurrently with pancreatic resection, resulting in a diagnosis of NTS [17, 26, 30]. These reports suggest that in cases of transgastric EUS‐FNA followed by DP for pancreatic cancer, surveillance for NTS should include monitoring of tumor marker trends, CT imaging, and EGD for approximately 3 years after surgery. Further studies are required to establish the optimal surveillance strategy.

In a national survey, 25 of 38 patients with NTS after EUS‐FNA for pancreatic cancer underwent resection of the NTS, whereas the remaining 13 did not. The median survival was 51.9 months for patients who underwent NTS resection, compared with 26.2 months for those who did not, suggesting a survival benefit associated with resection [31]. These results indicate that resection of NTS may improve prognosis when feasible.

At present, no established method exists for preventing NTS. Although 22‐gauge needles are commonly used during EUS‐FNA in reported cases of NTS, occurrences have also been described with 25‐gauge needles [16, 18, 21, 29], and the relationship between needle gauge and NTS incidence remains unclear. Reducing the number of needle passes may potentially decrease the risk. The use of an FNB needle to obtain sufficient tissue with fewer passes may also mitigate the risk. In addition, washing the puncture needle with sterile saline after each pass has been proposed as a preventive measure. When transgastric EUS‐FNA is performed, partial gastrectomy, including the puncture site, may be considered.

We present a case of NTS diagnosed 12 months after transgastric EUS‐FNA and DP for pancreatic tail cancer. This case highlights the importance of recognizing the risk for NTS in patients with pancreatic cancer who undergo transgastric EUS‐FNA and emphasizes the necessity of vigilant surveillance and appropriate management.

## Author Contributions

**Hidetaka Kuroda**: data acquisition, analysis, interpretation of the data, and drafting of the manuscript. **Takuji Iwashita**: literature review and critical revision of the manuscript. **Yosuke Ohashi**, **Shota Iwata**, **Ryuichi Tezuka**, **Shinya Uemura**, **Katsutoshi Murase**, **Nobuhisa Matsuhashi**, **Tatsuhiko Miyazaki**, and **Masahito Shimizu**: data interpretation and manuscript review.

## Funding

The authors have nothing to report.

## Conflicts of Interest

Prof. Takuji Iwashita is a Deputy Editor‐in‐Chief of DEN Open.
